# Self-Propagating Synthesis and Characterization Studies of Gd-Bearing Hf-Zirconolite Ceramic Waste Forms

**DOI:** 10.3390/ma12010178

**Published:** 2019-01-07

**Authors:** Kuibao Zhang, Dan Yin, Kai Xu, Haibin Zhang

**Affiliations:** 1 State Key Laboratory of Environment-friendly Energy Materials, Southwest University of Science and Technology, Mianyang 621010, China; yindan@swust.edu.cn; 2 State Key Laboratory of Silicate Materials for Architectures, Wuhan University of Technology, Wuhan 430070, China; kaixu@whut.edu.cn; 3 Sichuan Civil-Military Integration Institute, Mianyang 621010, China; 4 Institute of Nuclear Physics and Chemistry, China Academy of Engineering Physics, Mianyang 621900, China

**Keywords:** self-propagating, nuclear waste, zirconolite, actinide, aqueous durability

## Abstract

Synroc is recognized as the second-generation waste matrice for nuclear waste disposal. Zirconolite is one of the most durable Synroc minerals. In this study, Gd and Hf were selected as the surrogates of trivalent and tetravalent actinide nuclides. Gd-bearing Hf-zirconolite (Ca_1−x_Hf_1−x_Gd_2x_Ti_2_O_7_) ceramic waste forms were rapidly synthesized from a self-propagating technique using CuO as the oxidant. The results indicate that Gd can concurrently replace the Ca and Hf sites. However, Gd_2_O_3_ could not completely be incorporated into the lattice structure of zirconolite when the x value is higher than 0.8. The aqueous durability of selected Gd-Hf codoped sample (Hf-Gd-0.6) was tested, where the 42 days normalized leaching rates (*LR_i_*) of Ca, Cu, Gd and Hf are measured to be 1.57, 0.13, 4.72 × 10^−7^ and 1.59 × 10^−8^ g·m^−2^·d^−1^.

## 1. Introduction

Due to the main contribution of minor actinides (Np, Am, Cm) to the long term radiotoxicity of high-level nuclear wastes (HLW) recovered from spent fuel reprocessing, the separation of the actinide nuclides and their immobilization in durable matrices have been of prime importance [1,2]. A large number of fundamental and engineering orientated studies have been launched in several countries (France, Japan, Russia, et al.) to explore the feasibility of highly stable matrices, such as ceramics, glass-ceramics or glasses [3,4,5,6,7,8,9,10,11,12,13,14,15]. Among these host materials, borosilicate glass has been proved as a desirable matrice for large-scale applications [4,11]. However, the low solubility of minor actinides in glass matrix and the relatively low thermal stability of glass are the major limitations for the disposal of actinide-rich wastes [10,16]. Alternatively, Synroc has been proposed as a potential matrice for HLW immobilization by Ringwood et al. [17]. Synroc is mainly composed of multiple titanate mineral phases, such as zirconolite (CaZrTi_2_O_7_), pyrochlore (A_2_B_2_O_6_X), perovskite (CaTiO_3_), hollandite (BaAl_2_Ti_6_O_16_), rutile (TiO_2_), spinel (AB_2_O_4_), et al. These mineral phases have accommodated actinide elements in the natural environment for over tens of millions of years. According to the theory of isomorphism substitution, radioactive nuclides can be included into the lattice structure of above-mentioned mineral phases, which can significantly promote the waste loading and long-term stability [17,18,19,20,21,22,23].

Zirconolite, which is one of the most durable phases among Synroc minerals, has been extensively investigated as a ceramic matrice [24,25,26,27]. Zirconolite exhibits a layered structure, which is formed by the stacking layers of edge shared Ti-O polyhedra (TiO_6_ and TiO_5_) and layers of Ca^2+^ and Zr^4+^ ions [25,26,27,28]. Due to the composition and nature of substitution, zirconolite can transform into different polytypes like zirconolite-2M (monoclinic), zirconolite-3T (trigonal), zirconolite-3O (orthorhombic). The different forms of coordination make zirconolite structure capable of accommodating large cations like rare-earth, actinide and alkaline earth ions, as well as small cations like transition metal ions [28,29,30]. Moreover, zirconolite-base waste forms exhibit excellent performances in waste loading, aqueous durability, chemical flexibility, radiation resistance and existence of nature analogues, which make it a potential host phase for the immobilization of separated minor actinides [4,7,8].

In general, zirconolite-rich Synroc waste forms were mainly synthesized by traditional methods, such as liquid phase synthesis (hydroxide and sol-gel methods) and solid state reaction [31,32,33]. These approaches usually require a long-time sintering process under high-temperature and high pressure, which is time consuming and evokes the risk of nuclide volatilization. Muthuraman et al. have been proposed an alternative synthesis approach, self-propagating high-temperature synthesis (SHS), for the immobilization of nuclear waste [34]. Because of its special advantages [35], SHS technique has been considered as a candidate approach for environment protection, such as stabilization of radioactive and toxic wastes. In recent years, we have explored the rapid synthesis of zirconolite and pyrochlore based waste forms using SHS [36,37,38,39,40,41]. Quick pressing (QP) was also introduced to obtain highly densified samples. The results demonstrate that highly densified ceramic-based waste forms can be synthesized within several minutes using this SHS/QP technique.

As real actinides contained HLW is not available in laboratory, simulated actinide nuclides are widely employed in fundamental research. From the consideration of crystal chemistry and ionic radius [42], Gd and Ce were usually employed as the surrogates of trivalent and tetravalent actinide elements. From previous studies [43,44], the charge state of Ce is not stable as Ce^4+^ usually transforms to Ce^3+^ under high temperature sintering. Actually, Hf is a better surrogate of tetravalent actinides (especially Pu) over Ce as the charge state of Hf^4+^ is extremely stable. Hf exhibits similar density and solubility as Pu in vitreous waste forms. Hf can also partially or totally replace the Zr site of zirconolite (Hf-zirconolite, CaHfTi_2_O_7_) [45,46]. Gd and Hf are considered as a neutron poison for fission reactions because they have extreme high capture cross-sections of thermal neutron [47]. Thus, the Gd-bearing Hf-zirconolite waste forms possess high critical safety when loaded with fissile actinide isotopes of ^239^Pu and ^235^U. In this study, Hf-zirconolite was rapidly prepared from an SHS/QP technique using CuO as the oxidant. The Zr site was totally replaced by Hf with chemical composition of CaHfTi_2_O_7_. On this basis, Gd_2_O_3_ was introduced as the surrogate of trivalent actinides, which was designed to concurrently occupy the Ca and Hf sites of Hf-zriconolite. The phase composition, crystal structure, site occupancy and microstructure of the Gd-bearing samples were investigated. In addition, the aqueous durability was evaluated using the standard MCC-1 leaching test [48].

## 2. Materials and Methods

Analytical grade CuO, CaO, Ti, TiO_2_, ZrO_2_, as well as high purity Gd_2_O_3_ and HfO_2_ (purity ≥ 99.9 wt. %), were purchased as the raw materials. Firstly, the Hf-zirconolite was prepared according to the following chemical equation:

6CuO + 2CaO + 3Ti + TiO_2_ + 2HfO_2_ = 2CaHfTi_2_O_7_ + 6Cu (1)

After that, a series of compositions with stoichiometry as Ca_1−x_Hf_1−x_Gd_2x_Ti_2_O_7_ (x = 0.2, 0.4, 0.6, 0.8 and 1.0, named as Hf-Gd-0.2, Hf-Gd-0.4, Hf-Gd-0.6, Hf-Gd-0.8 and Hf-Gd-1.0) were synthesized from this SHS technique. The designed SHS reactions were conducted as follows:

6CuO + 2(1 − x)CaO + 3Ti + TiO_2_ + 2(1 − x)HfO_2_ + 2xGd_2_O_3_ = 2Ca_1−x_Hf_1−x_Gd_2x_Ti_2_O_7_ + 6Cu (2)

The weight percentages of raw materials are listed in Table 1. About 20 g reactants were completely homogenized using planetary ball milling. The mixed powders were then preformed into cylindrical pellets with dimension of Φ25 × 12 mm. The pressed pellets were then ignited and densified similarly to in our previous report [36]. Before pressure exertion, the reaction temperatures of all samples were measured by a W/Re 5/26 thermocouple located at the sample center.

The as-synthesized specimens were pulverized into fine powders, which were characterized by X-ray diffractometer (XRD; D/MAX-RB, Rigaku Corporation, Tokyo, Japan) with Cu Kα radiation to obtain the phase composition. The ignited samples were compressed by a quick pressing of 45 MPa with 60 s holding time after about 25–30 s delay of combustion. The obtained samples were then sliced and polished using different grades of emery paper and 0.5 μm diamond pastes. After cleaning and drying, the samples were subjected to further characterizations. Microstructure of the selected Hf-Gd-0.6 sample was typically observed using field-emission scanning electron microscopy (FESEM; Zeiss Ultra-55, Oberkochen, Germany) under 15 KV energy. The phase composition and elemental distribution were analyzed from the results of energy-dispersive X-ray spectrometer (EDX, ULTRA 55, ZEISS, Oberkochen, Germany) attached with the FESEM equipment. The chemical durability of Hf-Gd-0.6 sample was evaluated using standard MCC-1 leaching test. The specimen was sliced and grinded into dimension of 5.28 mm × 5.30 mm × 5.24 mm, which was suspended by a copper wire and immersed in 80 mL deionized water. Completely cleaned polytetra-fluoroethylene (PTFE) was utilized the leaching container. The leaching tests were carried out at 90 °C with durations of 1, 3, 7, 14, 21, 28, 35 and 42 days. The elemental concentrations of Ca and Cu in the leachates were obtained by inductively coupled plasma (ICP) analysis (iCPA 6500, ThermoFisher, Waltham, MA, USA), while Hf and Gd were collected by inductively coupled plasma-mass spectrometry (ICP-MS) analysis using an Agilent 7700× spectrometer (Santa Clara, CA, USA).

## 3. Results and Discussion

### 3.1. Combustion Temperature and XRD Analysis of the Hf-Zirconolite Sample

**Figure 1:**
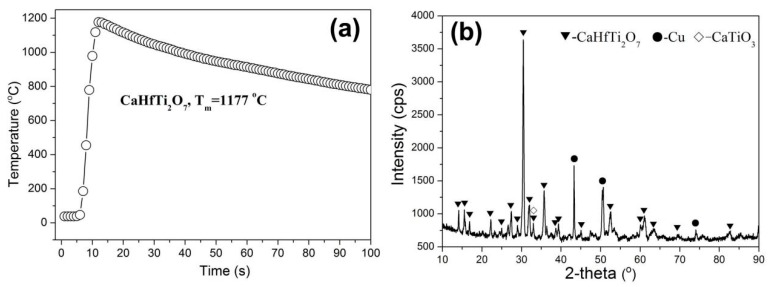
(**a**) Reaction temperature of the Hf-zirconolite sample, (**b**) XRD pattern of the Hf-zirconolite sample.

According to the previous research [45], Hf can totally replace the Zr site of zirconolite. In this experiment, we firstly testify the feasibility for the SHS preparation of Hf-zirconolite. The combustion experiment of the above-mentioned Equation (1) was conducted. The result demonstrates that the green body can be successfully ignited with self-sustaining reaction. The combustion lasts for about 10 s after ignition, which leads to a reaction speed of about 2–3 mm/s. The center temperature of this sample was measured as depicted in Figure 1a. The maximum temperature is 1177 °C and the temperature duration (≥1000 °C) is longer than 30 s. As there is heat dissipation during the combustion reaction and subsequent testing, the real temperature should be much higher than the measured one. This temperature is adequate and beneficial for subsequent compression as it is higher than the melting point of Cu (1083 °C). Figure 1b shows the XRD pattern of the obtained Hf-zirconolite sample, which indicates the phase composition mostly conforms to the original design. Hf-zirconolite and Cu demonstrate are the main phases with a trace of CaTiO_3_ phase. As no peaks correspond to HfO_2_, we can confirm that HfO_2_ has been completely incorporated into the Zr site of zirconolite. Because the Zr^4+^ and Hf^4+^ cations are in the same charge state and close ionic radius (0.72 Å for Zr^4+^ and 0.71 Å for Hf^4+^), they can mutually substituted under random proportion. This result testifies that Hf-zirconolite can be readily synthesized using the SHS method.

### 3.2. Reaction Temperature and Phase Composition of Gd-Bearing Hf-Zirconolite Samples

**Figure 2:**
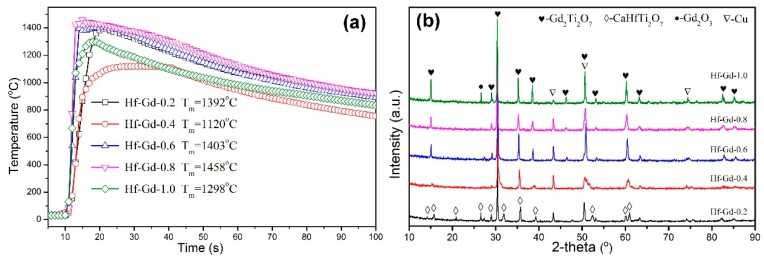
(**a**) Reaction temperatures, (**b**) XRD patterns of the Gd-bearing Hf-zirconolite samples with x values of 0.2–1.0.

The Gd-bearing Hf-zirconolite waste forms were subsequently synthesized. All the designed SHS reactions were successfully ignited and the combustions lasted for about 10 s after tungsten wire ignition. The center temperatures were collected and depicted in Figure 2a. There is not a trend of regularity for the temperature of Gd-bearing samples. The maximum temperatures of these five samples reach to 1392 °C, 1120 °C, 1403 °C, 1458 °C and 1298 °C as the x value is elevated from 0.2 to 1.0. Compared with the original Hf-zirconolite (1177 °C), the Gd_2_O_3_ doped samples exhibit much higher temperatures (except for the Hf-Gd-0.4 sample). This result reveals that the reactivity of Gd_2_O_3_ is higher than CaO or/and HfO_2_. Although the temperatures are not high, they are adequate and facilitate the subsequent densification process to get highly densified samples.

According to previous studies [29,31], the Ca and Zr site of zirconolite could be concurrently occupied by trivalent actinides. The phase compositions of Gd-bearing Hf-zirconolite samples were characterized with the XRD patterns presented in Figure 2b. It is distinctly demonstrated that there is a phase transformation from 2M-zirconolite to cubic pyrochlore as the x value is elevated. There are only Hf-zirconolite (CaHfTi_2_O_7_, PDF No. 84-0163) and Cu phases in the Hf-Gd-0.2 sample. Minor pyrochlore appears when the x value is 0.4. The pyrochlore phase demonstrates as the main phase when the x value is 0.6, which can be verified by the superlattice (100) diffraction peak at around 15°. This result is similar as the Nd-bearing zirconolite in the solid-state synthesized CaZrTi_2_O_7_-Nd_2_Ti_2_O_7_ system [31]. However, unreacted Gd_2_O_3_ is detected in the Hf-Gd-1.0 sample, which indicates that Gd could not totally substitute the Ca and Hf sites. This phenomenon may be related with the highly different ionic radius between Gd^3+^ (0.938 Å) and Hf^4+^ (0.71 Å). The maximum loading capacity of Gd_2_O_3_ is the Hf-Gd-0.8 sample, and only Gd_2_Ti_2_O_7_-based pyrochlore (PDF No. 73-1698) and Cu are demonstrated as the constituent phases in this sample.

### 3.3. SEM and EDX Analysis of the Gd-Doped Samples

**Figure 3:**
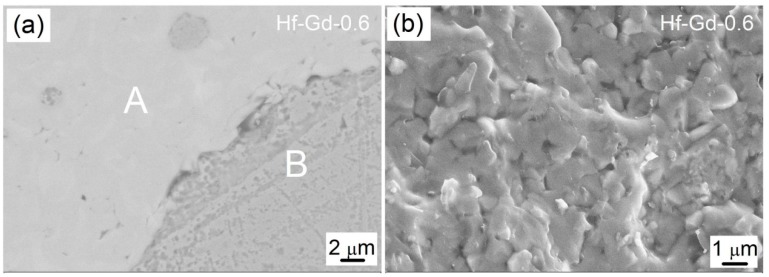
SEM images of the Hf-Gd-0.6 sample: (**a**) the polished surface, (**b**) the fracture surface.

The observed phase fields in the Ca_1−x_Hf_1−x_Gd_2x_Ti_2_O_7_ system were further supported by the SEM and EDX analysis. Typical back-scattered electron (BSE) image of the selected Hf-Gd-0.6 sample is shown in Figure 3a. No obvious pores can be observed in the surface image, which indicates this sample was well densified. Meanwhile, two different phases with distinct contrasts can be detected in the polish surface. The ceramic matrix phase is labeled as “A” and the metallic Cu phase is labelled as “B”. The Cu phase can be readily determined because it is segregated by a distinct boundary. The brightness of “A” district is obviously higher than “B”, which is attributed to the higher atomic number over Cu for Ca_1−x_Hf_1−x_Gd_2x_Ti_2_O_7_ phase. According to the XRD result, the ceramic matrix should be pyrochlore-based titanate with a small amount of zirconolite phase. Figure 3b presents the fracture surface of Hf-Gd-0.6 sample, which exhibits a dense microstructure with tightly contacted submicron sized grains. The grain boundary is not very clear in the polishing surface and fracture surface, which reveals the feature of combustion synthesis as the reaction speed is high and soaking time is short. There is no time for the formation of grain boundary and grain growth.

**Figure 4:**
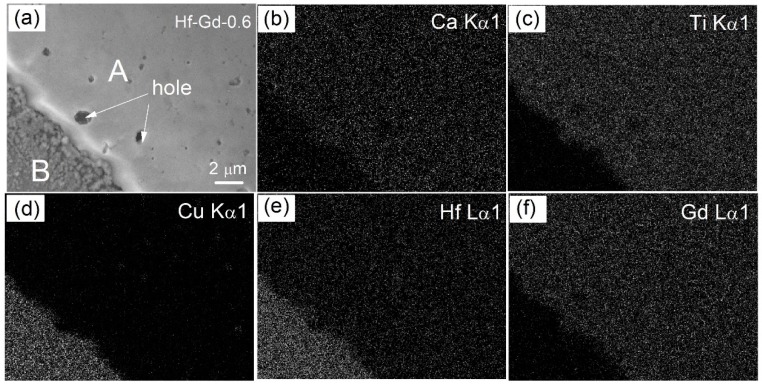
SEM-EDX mapping images of the Hf-Gd-0.6 sample: (**a**) representative BSE image, (**b**–**f**) elemental distribution of Ca, Ti, Cu, Hf and Gd elements.

Elemental EDX characterization was further conducted to determine the phase composition and elemental distribution of the typical Hf-Gd-0.6 specimen. The BSE and EDX mapping images are presented in Figure 4, where all the metallic elements of Ca, Ti, Hf, Gd and Cu are listed. The representative BSE image of Figure 4a supports the coexistence of “A” and “B” phases. Obviously, the “B” area must be Cu phase, which is testified by the EDX mapping image of Figure 4d. The “A” phase should be Ca_1−x_Hf_1−x_Gd_2x_Ti_2_O_7_ phase as the Ca, Ti, Gd elements are enriched in this area. This result conforms to the phase composition of XRD analysis. It’s worth noting that Hf not only appears in the matrix A area but also in the Cu phase. The enrichment of Cu and Hf elements is slightly overlapping in the “B” area. This phenomenon is strange as there is no peak corresponding to Hf or HfO_2_ in the XRD pattern. It may be attributed to the adjacent energy characteristic peaks of Cu and Hf in the EDX spectra (Hf: K_α_ = 8.040, K_β_ = 8.903, Cu: K_α_ = 7.898, K_β_ = 9.021).

**Figure 5:**
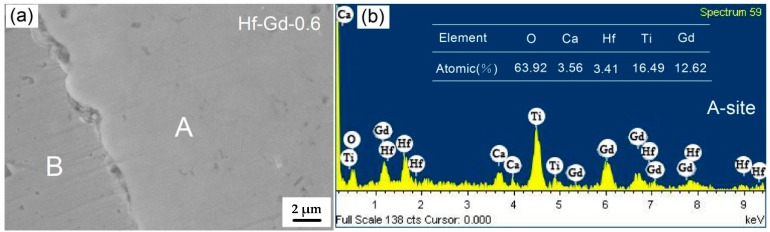
EDX spotting results of the Hf-Gd-0.6 sample: (**a**) representative BSE image, (**b**) elemental spotting analysis of the “A” region.

The EDX spotting analysis was further conducted to determine the chemical composition of the constituent ceramic phase, where the results are demonstrated in Figure 5. The EDX spotting analysis demonstrates that Hf has not been detected in the Cu phase. The EDX spotting image of “A” phase in Figure 5a is presented in Figure 5b. Similar as the EDX mapping results, the existence of Ca, Ti, Zr, Hf and O in the EDX spotting spectra indicates that the “A” phase is Gd and Hf doped pyrochlore phase. At least five points of “A” area were calculated to obtain the average elemental quantities as listed in Figure 5b. Based on this data, the chemical formulation of ceramic phase is calculated as Ca_0.39_Hf_0.37_Gd_1.38_Ti_1.80_O_7_. Compared with the designed formulation of Hf-Gd-0.6 sample (Ca_0.4_Hf_0.4_Gd_1.2_Ti_2_O_7_), the obtained ceramic phase is slightly deficient in Ti while rich in Gd. The Ca and Hf elements are very close to the designed values. This result testifies that the ceramic phase is in pyrochlore structure, where the Ca and Hf elements occupy the A site (Gd site in this study) of A_2_B_2_O_7_ pyrochlore.

### 3.4. Chemical Stability of the Hf-Gd-0.6 Sample

**Figure 6:**
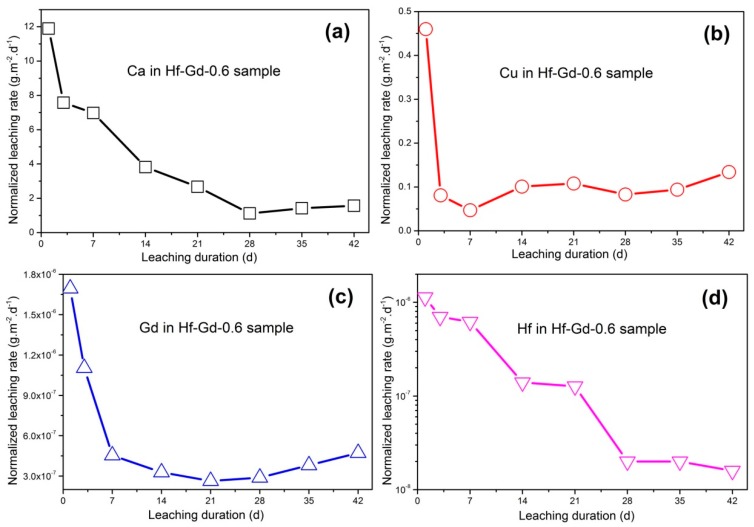
1–42 days normalized leaching rates of the Hf-Gd-0.6 sample: (**a**) element Ca, (**b**) element Cu, (**c**) element Gd, (**d**) element Hf.

The representative Hf-Gd-0.6 specimen was selected for the standard MCC-1 leaching test. The 1–42 days normalized elemental leaching rate of Ca, Cu, Gd and Hf are computed and depicted in Figure 6a–d. With the increase of soaking duration, all the normalized leaching rates firstly decrease in 1–7 days. However, the LR_Cu_ and LR_Gd_ exhibit slight ascension when the leaching time is prolonged (7 days for Cu and 21 days for Gd). Anyhow, the *LR_Ca_* and *LR_Cu_* values are 1.57 g·m^−2^·d^−1^ and 0.13 g·m^−2^·d^−1^ after 42 days. Gd and Hf are highly durable elements as shown in Figure 6c,d. Although there is a slight increase, the 42 days *LR_Gd_* value is as low as 4.72 × 10^−7^ g·m^−2^·d^−1^. The *LR_Hf_* value exhibits a congruent decrease tendency during 1–42 days leaching, where the leaching rate is 1.11 × 10^−8^ g·m^−2^·d^−1^ after 42 days. In this experiment, the leaching rate of Ca and Cu is comparable while Gd and Hf are even lower than Synroc waste forms prepared by hot pressing (HP) or hot isostatic pressing (HIP) [32,33]. The leaching rates are also significantly lower than borosilicate glass (about 1 g·m^−2^·d^−1^, 90 °C) [2,3,49].

## 4. Conclusions

In this study, Gd-bearing Hf-zirconolite (Ca_1−x_Hf_1−x_Gd_2x_Ti_2_O_7_) waste forms were rapidly synthesized from the SHS/QP method using CuO as the oxidant. Gd and Hf were employed as the simulates of trivalent and tetravalent actinides. The results indicate that Hf can totally replace the Zr site using this SHS process, and Gd can concurrently replace the Ca and Hf sites (Gd preferentially substitutes the Ca site). Gd_2_O_3_ could not completely be incorporated into the lattice structure of zirconolite when the x value is higher than 0.8. The aqueous durability of selected Hf-Gd-0.6 sample was tested, where the 42 days normalized leaching rates (*LR_i_*) of Ca, Cu, Gd and Hf are measured to be 1.57, 0.13, 4.72 × 10^−7^ and 1.59 × 10^−8^ g·m^−2^·d^−1^. These results demonstrate that the SHS/QP route is suitable for the preparation of zirconolite and pyrochlore based waste forms for HLW immobilization.

## Figures and Tables

**Table 1:** Weight percentage of the raw reactants for Gd-doped Hf-zirconolite samples.

Sample No.	Addictive Amount of Raw Materials (g)					
	CuO	CaO	HfO_2_	Ti	TiO_2_	Gd_2_O_3_
Hf-Gd-0.2	7.502	1.410	5.294	2.257	1.255	2.279
Hf-Gd-0.4	7.282	1.026	3.854	2.191	1.218	4.426
Hf-Gd-0.6	7.075	0.665	2.496	2.128	1.184	6.450
Hf-Gd-0.8	6.879	0.323	1.213	2.069	1.151	8.362
Hf-Gd-1.0	6.694	-	-	2.014	1.120	10.171
